# Moving around a Large City in Latin America: The Mobility Challenges Faced by Older Adults with Disabilities

**DOI:** 10.3390/ijerph182412984

**Published:** 2021-12-09

**Authors:** María-Eugenia Prieto-Flores, Mark W. Rosenberg

**Affiliations:** 1 National Council of Scientific and Technical Research (CONICET), Institute of Geography, National University of La Pampa, Santa Rosa 6300, Argentina; 2 Department of Geography and Planning, Queen’s University, Kingston, ON K7L 3N6, Canada; mark.rosenberg@queensu.ca

**Keywords:** aging, disability, urban environment, dependent mobility, age-friendly environments, Buenos Aires, Argentina

## Abstract

A growing body of research has shown that barriers in the urban environment can be disabling by reducing the ability of older people to manage independently in the community, but also because they can negatively affect health by limiting the possibilities to move outside the home. In this study, we ask how obstacles in the urban environment are associated with the need for help to go to places in the community. To respond to this question, we used the Annual Household Survey of the City of Buenos Aires, Argentina 2018, which had a specific questionnaire for people with disabilities. From this sample, we selected adults aged 65 years or older with difficulties in at least one of six domains: vision; hearing; upper and lower body mobility; cognition; self-care; and communication. The final sample consisted of 513 persons (weighted = 109,316). First, we conducted a principal component analysis identifying three factors from variables of obstacles to access and use the urban environment: transportation; outdoor spaces; and information. Second, through a logistic regression model, we observed a direct relationship between these factors and the need for help to move in the community, controlling for sociodemographic characteristics, health status, and number of disabilities. This paper provides evidence on the significance of improving urban spaces to reduce dependent mobility. In Latin America, cities still face many challenges in becoming more age-friendly.

## 1. Introduction

Urban environments can be disabling by reducing the ability of older people to function independently, autonomously, and safely in the community, but also because they can negatively affect health by limiting the opportunities for moving and doing activities outside the home. As reflected in Wiles and Allen [1], physically disabling environments and socially hostile attitudes towards old age are examples of disabling geographies.

Growing older, vulnerability to the physical and social environment increases, which according to the environmental characteristics, may restrict or favor the development of daily activities [2,3]. These environments not only influence disability, but also dependence, and the distance between one and the other may vary with improvements in the environment, including the removal of barriers, which contributes to greater autonomy for people with disabilities [4].

Environmental perspectives on aging have been fundamental in demonstrating the processes of interaction between the person and the environment, thanks to the contribution of various disciplines, such as psychology, geography, sociology, architecture, and occupational therapy [5]. The rich work of Lawton and colleagues, in particular, their Ecological Model of Aging [6], was essential in introducing environmental factors into research on health and aging, developing the concept of the person-environment fit to explain the relationship and the search for balance that occurs between the abilities of older people and the demands of their environments, as they experience changes in functional capacities [7].

In its World Report on Aging and Health, WHO [8] defines healthy aging as the process of developing and maintaining the functional ability that contributes to wellbeing in older age. It also indicates that this functional ability depends on the intrinsic capacity of the person (combination of physical and mental capacities), on the characteristics of the environment in which they live (physical, social and political, from the micro to the macro level), and on the interactions between the two. The distribution of functional capacity among older adults is not random, but responds, in a large extent, to the cumulative effect of social inequalities in health throughout the life course [4,9,10].

Supportive environments are critical across the lifespan to promote healthy living, which is a key objective of the movement of age-friendly environments. An age-friendly city or community is one in which the policies, services, and structures related to the physical and social environment contribute to enable older people to enjoy good health, live in security, and continue to participate in society [11,12]. Such a community is a good place to grow old, helping people to remain independent for as long as possible, providing protection and care when needed, and respecting autonomy and dignity of older adults [13]. Age-friendly environments influence personal mobility, health outcomes, activity and social participation, and quality of life in old age [12,14,15,16,17,18].

The core dimensions of friendly environments, according to the experience of older people in cities from different countries of the world, are: outdoor spaces and buildings; transportation; housing; social participation; respect and social inclusion; civic participation and employment; communication and information; and community and health services [12]. These eight areas overlap and interact.

Specifically, the ability and opportunity to move outside the home play an important role in older adults’ independent living and social participation [19]. The work of Bryant, Corbett, and Kutner [20] presents a model of healthy aging based on the perception of older people with different health reported statuses. In this model, healthy aging is represented by the ability to go and do something meaningful, which includes something worthwhile and desirable to do, balance between abilities and challenges, personal attitudinal characteristics, and appropriate external resources. For example, accessible transportation can complement the declining ability of older adults to maintain their independence and continue their activities.

Independence and mobility are based on relations with, and dependencies on, bodies, technologies, infrastructures, social networks, and social conditions [21]. Reasons for decreasing out-of-home mobility reflect personal and environmental circumstances, for example, declining health, insufficient money, having no companion, and difficulties using transport and environmental barriers [19]. In turn, mobility restrictions in old age may have health consequences related to access to food stores and health services, physical activity, and frequency of social contacts [22]. Environment, mobility, health, and disability are closely related, and influence the diverse experiences and trajectories of aging in cities.

Hallgrimsdottir and Ståhl [16] longitudinally examined the long-term impacts of improvements in the outdoor environment on an aging population, and found that walking can be facilitated for people who use mobility devices, and people with functional limitations. This has implications related to the role of the environment on independent mobility in older people with disabilities, which has not been sufficiently studied.

The capacity to provide adequate resources and environments to help older people to move safely is unequal among, and within, cities. The implementation of age-friendly policies has been compromised by pressures related to urban development, widening inequalities within urban settings, and the impact of budget cuts, especially in low-income communities [23].

In Latin America, the characteristics of the physical and social environment of the cities contribute in many cases to increase the vulnerability of older people, and governmental solutions are often insufficient and far from the needs of this population [24]. Scientific works on the relationship between aging persons and the urban environment are still limited in the region, despite the major social, health, and residential challenges posed by urban demographic aging [25].

In Argentina, 11 cities, including the capital, are part of the global network for the Age-Friendly Cities Project, which focuses on the lived experience of older people. Tordo and Gascón [26] summarize the coincidences in the diagnoses of all these cities, elaborated by focus groups formed by older people. The coincidences include difficulties related to insecurity, broken sidewalks, lack of maintenance of green spaces, inadequate public transportation due to the routes and characteristics of the vehicles, noncompliance with basic traffic regulations, inaccessible buildings, and problems related to health services. The provision of public services, and the maintenance of the cities present some deficiencies that negatively affect the quality of life of older adults [27].

As in many Latin American countries, in Argentina, socioeconomic and human development has followed different trajectories in its various geographic regions and social strata, which have had an impact on demographic aging [27]. The older population in Argentina has a very heterogeneous socio-spatial distribution. As expected, the most aged regions of the country are those with the highest socioeconomic level, and the lowest prevalence of disability, controlling for age effect [28]. Likewise, territorial inequalities have been identified in life expectancy free of permanent limitations, where the City of Buenos Aires represents the most advantageous situation [29]. However, inequalities are observed from the macro to the micro scale, as there are important intraurban socio-spatial variations in the risk of death in this city [30]. Considering the entire metropolitan area, life expectancy at birth can vary on average by more than 5 years, depending on the place of residence, although these gaps are smaller than those observed in some other large cities in Latin America [31].

In the context of this urban space and the framework presented, the objective of this study is to analyze in the older population with disabilities in the City of Buenos Aires, how obstacles to access and use the urban environment are associated with the need for help by another person to move in the community. Specifically, we consider self-reported information regarding obstacles and problems in three domains of age-friendly environments: transportation; outdoor spaces; and information. We hypothesize that the obstacles and problems in the three domains reduce independent mobility in the city.

## 2. Materials and Methods

### 2.1. Study Sample

This study was based on the Annual Household Survey of the City of Buenos Aires, Argentina, which provides information on the socioeconomic situation of the population, their households, and their dwellings (database available at https://www.estadisticaciudad.gob.ar/eyc/?p=105620 (accessed on 8 December 2021)). The survey data are cross-sectional, and are collected between October and December each year. It is a proportional stratified probability sample, and is carried out in two stages in dwellings in which all the households are surveyed. The sample is designed on the basis of two sampling frames: one corresponding to general private dwellings, and the other comprising dwellings in informal settlements. The sample is representative of the total population of the City of Buenos Aires and each of their communes, and weights are provided to adjust the sample data to the reference population.

The 2018 round of the survey included a specific module for people with disabilities, mainly identified as those who reported that they had a lot of difficulty or could not do certain activities because of a health problem, in accordance with some of the recommendations of the Washington Group on Disability Statistics [32]. Those difficulties belong to six functional domains: vision; hearing; upper and lower body mobility (walking or climbing steps, using hands and fingers); cognition; self-care; and communication. Given that disability is a dynamic process in which the characteristics of individuals and their environment interact, the questionnaire allows the analysis of the barriers and obstacles for activities of daily living. The module was carried out with the guidance of the Commission for Full Participation and Inclusion of People with Disabilities of the City of Buenos Aires.

Of the total sample (unweighted: 14,497; weighted: 3,067,990), adults aged 65 years old or more were 16.19%, and from this group, we selected those with disability in at least one of the six mentioned domains who responded to the disability module, representing 22.00% of the older population (unweighted: 513; weighted: 109,316).

### 2.2. Measures

To meet the objective of analyzing how obstacles are associated with the need for help to move in the City of Buenos Aires, the dependent variable was obtained from the question: “Because of your disability, do you need help regularly by another person to go to places and go out to the community?”, with response options “yes” or “no”.

The focal independent variables were derived from nine questions that asked the interviewees with disabilities if they found problems or obstacles in accessing or using the bus, subway, train, ramps, sidewalks, parks and squares, information and signage, information screens, and traffic lights (yes/no answers).

To control for the effect of sociodemographic and health characteristics in the analyses, the following variables were included: sex; age (continuous); education level in two groups (primary or lower, secondary or higher); homeowner (yes/no); living alone (yes/no); self-perceived health status (with values from 1, “very bad”, to 6, “excellent”); and number of disabilities (1–5).

### 2.3. Data Analysis

We first described the sample of this study, and performed bivariate analyses using the Mann–Whitney U Test for non-parametric scale variables, and the χ2 test for categorical ones. Second, we conducted a principal component analysis to group intercorrelated variables of the nine types of obstacles to access and use the urban environment, obtaining three factors. We applied this method following a variable reduction technique, in which a small set of principal components are extracted from many variables to provide a summary that explains most of the variance observed in all the initial variables. The principal components extract the latent structure of the original variables. Third, we applied a logistic regression model to analyze the relationship between these factors, and the need for help to go to places in the community. We estimated frequencies using the weighted and unweighted sample, but bivariate and multivariate analyses were based on unweighted data to avoid inflated statistical significance by using the relatively small sample size of people with disabilities. IBM SPSS statistical program (version 24.0.0, IBM Corp., Armonk, NY, USA) was used in all analysis steps.

## 3. Results

### 3.1. Characteristics of the Studied Population

According to the Annual Household Survey 2018, the disability prevalence determined by those who reported having a lot of difficulty or that they could not do activities at all in at least one functional domain is estimated as 201,406 people (7.1%) within the population aged more than 5 years old who live in the City of Buenos Aires, and 113,027 (22.8%) among older adults aged 65 years or more.

Table 1 presents the studied population composed of older adults with disabilities who responded to the disability module of the survey. The frequencies based on the unweighted sample are described below, according to sociodemographics, health characteristics, and obstacles in the urban environment, as well as the distribution of the need for help to go to places and go out in the community with respect to these variables.

The mean age was 79.3 years old, and the percentage of women almost doubled the proportion of men in this population: 63.9% vs. 36.1%, respectively. The number of people who did not complete studies higher than primary school was 32.2%, those who were not homeowners represented 21.7%, and older adults who lived alone were 35.1% of the older population. Perceived health status on the scale from 1 (very bad) to 6 (excellent) showed a mean value of 3.5, and the mean number of disabilities was 1.7. More than a half of the studied population indicated problems or obstacles in accessing or using public transport (bus: 61.6%, subway: 55%, train: 51.4%). In relation to outdoor spaces, 31.6% of the people surveyed indicated problems or obstacles related to ramps, 52.2% to sidewalks, and 32.7% in parks and squares. The problems of access to, and the use of, information and signage were reported by 20.1%, information screens by 16.4%, and traffic lights by 17.9%.

Almost half of the interviewed older people with disabilities needed help to go to places and go out in the community (47.5%). The distribution of this variable showed significant differences according to the sociodemographic, health, and urban obstacles characteristics, as can be seen in Table 1. Compared to older people who did not need help, those who depended on help were, to a greater extent, the oldest, women, persons without education higher than primary school, and those who were not homeowners. As expected, worse perceived health status, and a higher number of disabilities were related to the need for help. In contrast, no significant differences were found in the proportion of people who depended on help between the group that lived alone and the group that lived with others. Finally, most of the people who needed help identified problems or obstacles in accessing or using public transport, outdoor spaces, and information in the urban environment.

### 3.2. Urban Environmental Factors

The principal component analysis allowed us to group the nine interrelated variables of problems and obstacles in the urban environment into three factors. In accordance with the eigenvalues, which represent the variance accounted by each factor, we retained those greater than 1. Varimax rotation was applied to enhance the interpretation of the components. To test if the data were suitable for factor analysis, we applied Kaiser–Meyer–Olkin and Bartlett tests. The first showed good adequacy (0.84), and the second allowed us to reject the null hypothesis that the variables were uncorrelated (*p* < 0.001).

Table 2 shows the results of this analysis. The relationship of each variable to the underlying factor is expressed by the factor loadings, among which, those greater than 0.7 were retained. The obtained three factors explained 81.5% of the variance. The first one represents the variables related to problems or obstacles accessing or using transportation, “bus”, “subway”, and “train”; the second factor groups “information and signage”, “information screens”, and “traffic lights”; and the last one represents outdoor spaces variables, “ramps”, “sidewalks”, and “parks and squares”. The communalities showed high variables variance explained by the factors.

In addition, we applied a validation analysis of the original urban environmental variables, using Cronbach’s alpha test with the study sample. All the results showed high reliability levels. Reliability analysis of the original nine variables: Cronbach’s α = 0.881; transportation variables = 0.934; outdoor spaces variables = 0.839; information variables = 0.866.

### 3.3. Relationship between Urban Environmental Factors and the Need for Help to Move in the Community

The factor scores obtained from the principal component analysis were used to examine the role of the urban environmental obstacles, and the need for help to go to places in the community among older adults with disabilities in the City of Buenos Aires. Table 3 shows the results of the logistic regression model, including odds ratios (OR) with 95% confidence intervals (CI) and their significance level. The model correctly classifies 73.33% of the cases, and is statistically significant (Omnibus χ2 = 185.35, *p* < 0.001), and Nagelkerke’s R^2^ represents 0.407.

The need for help varies significantly according to demographic characteristics, number of disabilities, and the urban environmental factors. Thereby, the probability of depending on help increases with age (OR: 1.04, CI: 1.01–1.07), among women compared to men (OR: 1.62, CI: 1.01–2.60), and with the number of disabilities (OR: 1.52, CI: 1.15–2.00). The three factors representing urban environmental obstacles were found to have a highly significant independent effect on the need for help to move in the community: transportation (OR: 2.0 CI: 1.61–2.49); outdoor spaces (OR: 1.68, CI: 1.30–2.16); and information (OR: 1.89, CI: 1.52–2.34).

## 4. Discussion

The lack of environmental measures from existing datasets is one of the difficulties in conducting rigorous research on age-friendly communities [33], which is especially true in studies targeting older people with disabilities, despite their greater vulnerability to the limitations of the physical–social environment. In the present study, we analyzed the module for people with disabilities of the Annual Household Survey of the City of Buenos Aires 2018, which allowed us to investigate perceived urban obstacles and their effect on the need for assistance to move around in the community among older adults with disabilities.

Through principal component analysis, we obtained three factors based on nine variables related to problems or obstacles in accessing or using the bus, subway, train, ramps, sidewalks, parks and squares, information and signage, information screens, and traffic lights. These variables were correctly grouped according to three central dimensions of age-friendly environments: transportation; outdoor spaces; and information; which we used as key independent variables in our logistic regression model. The results of this model suggest that these three factors of problems and obstacles in the urban environment may reduce the probability of independent mobility of older people with disabilities, controlling for sociodemographic characteristics, health status, and number of disabilities.

Overall, studies on the association between the urban environment and mobility have shown that environmental characteristics can impact health, disability, social life, and wellbeing. Our research has contributed to examining the role of urban obstacles on independent mobility, which, to our knowledge, has not been sufficiently assessed.

Out-of-home mobility is a key representative form of person–environment interaction, involving the person, modes of transport, and the physical–social environment, all of which interact with each other [34]. Yen et al. [35], in their review on the impact of the built environment on older adults’ mobility, found that perception of safety plays a key role in mobility decisions, which is especially evident when experiencing functional limitations. It is possible that the lack of safety experienced reduces the possibility of moving in the community without assistance from another person.

In relation to transportation, there are numerous types of obstacles that older people encounter on public buses, related to access, distance to stops, distance from the curb, presence of steps, speed of driver take-off and stop, lack of bus shelters, and concern for personal safety [36]. Difficulties in using public transportation can reduce the community mobility of older adults, and negatively affect their social life and health [37]. In our study, we found that barriers to accessing or using the train, bus, and subway translated into increased likelihood of mobility dependence among older adults with disabilities.

As for outdoor spaces, previous studies have shown that walking difficulties reported by people with severe disabilities can be significantly increased among those living in neighborhoods where streets and sidewalks are in poor or fair condition [38]. This greater difficulty in moving around in the community, imposed by the characteristics of the environment, can lead to the need for assistance, as our work suggests, with obstacles on ramps, sidewalks, parks, and squares. If older people can feel comfortable and safe in public spaces, their willingness to spend time outdoors will increase [39]. Outdoor green spaces, in particular, can play an important role in promoting active aging through physical activities, social relationships, and community participation [40].

The ability to access urban services and facilities, and to meet and connect with people is not only diminished by obstacles in transportation and outdoor spaces, but also by barriers in communication and information, mainly in the visual and auditory presentation of information [12]. According to our findings, problems or obstacles related to the use of, or access to, information and signage, information screens, and traffic lights affect independent mobility in older people with disabilities.

Our work also allowed us to identify those groups of people with disabilities that, according to their personal characteristics, present a higher risk of dependent mobility: women; the most elderly; and those with greater disability; which is in line with previous studies that confirm that these groups are more vulnerable to the characteristics of the environment, and have a higher probability of mobility disability [34,41,42].

With regard to methodological issues, there are some considerations worth mentioning. The data used were self-reported. Future studies should include objective measures of the built environment, and complement the subjective approach. Ryan and Pereira [43] compared calculated and self-reported accounts to measure accessibility among older people, and found that when only “objective” indicators are taken into account, accessibility levels tend to be overestimated, and inequalities in accessibility are underestimated. This could be particularly important in unequal spaces, such as those of Latin American cities. Another methodological limitation of our work is that within the group of people who reported needing help to move around in the community, we do not know the proportion of those who were unable to do so independently, even in accessible environments. In accordance with the cross-sectional data of our study, we cannot measure the directionality of the observed relationships, but as stated in the introduction, personal functional abilities and the environment interact in old age. On the one hand, as people age and their functional limitations increase, they may perceive urban obstacles to a greater extent. But on the other hand, environmental barriers negatively impact the different dimensions of older adults’ health, which, in turn, may reduce their ability to move and remain active. Finally, it is important to note that mobility disability does not necessarily translate into mobility dependence if the urban environment is accessible.

The City of Buenos Aires has improved in certain aspects its physical environment towards the inclusion of people with disabilities, but there are still several restrictions for them to move independently and safely [44,45]. The existing regulations in Argentina have followed the international movement towards the progressive recognition of the rights of people with disabilities, but at the same time, it is well known that, in practice, there is a high level of non-compliance with the laws [46]. In the sample we analyzed, represented by older adults with disabilities, more than half reported encountering problems or obstacles related to public transportation and sidewalks. There is still a long way to go to properly remove physical barriers, and provide good maintenance for the current accessibility features in Argentina [46].

Within the group of older people with disabilities analyzed, some need special attention, as they are more likely to depend on help to move around outdoors: women; the most elderly; and those with multiple and severe disabilities. Persons with these characteristics, living in an unfriendly environment with limited social networks, may be at greater risk of confinement and social isolation.

## 5. Conclusions

This paper provides empirical evidence on the role of the urban environment in independent mobility in a large city of a middle-income country. This new information has implications for practices related to determinants that can be modified through urban policies and community planning that promote accessible and inclusive spaces to facilitate mobility, and prevent dependence, favoring social integration of people of all ages in the community.

This work also has implications related to care provision. The sustained increase in poverty and inequality experienced in Argentina is forecast to increase disability in old age [10], which, in turn, may translate into higher levels of dependency in a country where long-term care is mainly assumed by families, especially women, many of whom are older [47]. In this context, the obstacles of the physical environment become particularly relevant, as they impact the mobility of a growing vulnerable older population.

Future research could focus on analyzing spatial variations and patterns in urban accessibility to identify those places that require greater effort to be age-friendly. Another direction of research could be oriented to the study of factors associated with the availability of assistance for older people with mobility dependence to access the community.

## Figures and Tables

**Table 1:** Characteristics of the studied population of older adults with disability, and bivariate relations with the need for help to move in the community.

Variables	Weighted Sample		Unweighted Sample		Need Help Move in the Community ^b^				Significance Level
					No		Yes		
	*n*	%	*n*	%	*n*	%	*n*	%	
	109,316	100	513	100	269	52.5	243	47.5	
*Sociodemographic*									
Age (65–99) (M ± 1 SD) ^a^	79.8 ± 8.11		79.3 ± 8.3		77.6 ± 7.5		81.2 ± 8.6		*p* < 0.001 (U)
Sex									*p* < 0.001 (χ2)
Women	72,498	66.3	328	63.9	151	46.2	176	53.8	
Men	36,818	33.7	185	36.1	118	63.8	67	36.2	
Education level									*p* = 0.001 (χ2)
Primary or lower	31,785	29.1	165	32.2	69	42.1	95	57.9	
Secondary or higher	77,531	70.9	348	67.8	200	57.5	148	42.5	
Homeowner									*p* = 0.004 (χ2)
No	22,204	20.4	111	21.7	45	40.5	66	59.5	
Yes	86,852	79.6	401	78.3	224	56.0	176	44.0	
Living alone									*p* = 0.572 (χ2)
No	64,702	59.2	333	64.9	178	53.5	155	46.5	
Yes	44,614	40.8	180	35.1	91	50.8	88	49.2	
*Health*									
Self-perceived health status (1–6)	3.5 ± 0.9		3.5 ± 0.9		3.6 ± 0.9		3.3 ± 0.8		*p* < 0.001 (U)
Number of disabilities (1–5)	1.7 ± 1.0		1.7 ± 0.9		1.3 ± 0.7		2.0 ± 1.1		*p* < 0.001 (U)
*Problems or obstacles*									
*Transportation*									
Bus									*p* < 0.001 (χ2)
No	41,136	37.6	197	38.4	152	77.6	44	22.4	
Yes	68,180	62.4	316	61.6	117	37.0	199	63.0	
Subway									*p* < 0.001 (χ2)
No	49,127	44.9	231	45.0	175	76.1	55	23.9	
Yes	60,189	55.1	282	55.0	94	33.3	188	66.7	
Train									*p* < 0.001 (χ2)
No	53,388	49.0	249	48.6	187	75.4	61	24.6	
Yes	55,622	51.0	263	51.4	82	31.2	181	68.8	
*Outdoor spaces*									
Ramps									*p* < 0.001 (χ2)
No	74,177	67.9	351	68.4	228	65.1	122	34.9	
Yes	35,139	32.1	162	31.6	41	25.3	121	74.7	
Sidewalks									*p* < 0.001 (χ2)
No	51,035	46.7	245	47.8	180	73.8	64	26.2	
Yes	58,281	53.3	268	52.2	89	33.2	179	66.8	
Parks and squares									*p* < 0.001 (χ2)
No	73,561	67.3	345	67.3	225	65.4	119	34.6	
Yes	35,755	32.7	168	32.7	44	26.2	124	73.8	
*Information*									
Information and signage									*p* < 0.001 (χ2)
No	87,061	79.6	410	79.9	246	60.1	163	39.9	
Yes	22,255	20.4	103	20.1	23	22.3	80	77.7	
Information screens									*p* < 0.001 (χ2)
No	90,012	82.3	429	83.6	254	59.3	174	40.7	
Yes	19,304	17.7	84	16.4	15	17.9	69	82.1	
Traffic lights									*p* < 0.001 (χ2)
No	88,784	81.2	421	82.1	251	59.8	169	40.2	
Yes	20,532	18.8	92	17.9	18	19.6	74	80.4	

Note: ^a^ Mean and standard deviation (M ± SD) of the quantitative variables. ^b^ Unweighted sample. Weighted proportions of the variable are: No = 54,960 (50.36%), Yes = 54,181 (49.56%). U = Mann–Whitney U Test. χ2 = Chi-Squared Test. Any *p* < 0.05 was considered significant.

**Table 2:** Principal component analysis for urban environmental obstacles.

Problems or Obstacles to Accessing or Using	Components and Loadings			Communalities
	Transportation	Information	Outdoor Spaces	
Bus	0.892	0.114	0.203	0.850
Subway	0.906	0.149	0.249	0.905
Train	0.900	0.169	0.232	0.892
Information and signage	0.098	0.896	0.193	0.850
Information screens	0.134	0.809	0.178	0.704
Traffic lights	0.163	0.859	0.204	0.806
Ramps	0.187	0.310	0.801	0.772
Sidewalks	0.350	0.076	0.786	0.745
Parks and squares	0.186	0.272	0.839	0.812
Percentage variance explained	51.42	18.72	11.37	81.51

Note: Extraction method: principal component analysis; rotation varimax with Kaiser normalization. Kaiser–Meyer–Olkin measure of sampling adequacy: 0.84. Bartlett’s test of sphericity: χ2 = 3100.40 (*p* < 0.001). Factor loadings greater than 0.7 are shown in gray.

**Table 3:** Logistic regression model on the probability of needing help to move in the community.

Variables	Exp (β)	95 % IC Exp (β)	
		Lower	Upper
Age	1.04	1.01	1.07 *
Sex *(reference: men)*			
Women	1.62	1.01	2.60 *
Education level *(reference: secondary of higher)*			
Primary or lower	1.23	0.78	1.94
Homeowner *(reference: yes)*			
No	1.60	0.94	2.71
Living alone *(reference: no)*			
Yes	0.97	0.60	1.54
Self-perceived health status *(1–6)*	0.90	0.69	1.17
Number of disabilities *(1–5)*	1.52	1.15	2.00 **
Transportation factor	2.00	1.61	2.49 ***
Outdoor spaces factor	1.68	1.30	2.16 ***
Information factor	1.89	1.52	2.34 ***

Note: Significance level: * *p* < 0.05; ** *p* < 0.01; *** *p* < 0.001. Exp (β) = odds ratio; CI = confidence interval. Percentage of correct prediction = 73.33%. Omnibus χ2 = 185.35, *p* < 0.001. Nagelkerke’s R^2^ = 0.407.

## Data Availability

The data presented in this study are openly available in the Annual Household Survey of the City of Buenos Aires, Argentina, 2018 at https://www.estadisticaciudad.gob.ar/eyc/?p=105620 (accessed on 8 December 2021).
